# Long-term effects of bilateral subthalamic nucleus stimulation on sleep in patients with Parkinson’s disease

**DOI:** 10.1371/journal.pone.0221219

**Published:** 2019-08-27

**Authors:** Ji-Hyun Choi, Han-Joon Kim, Jee-Young Lee, Dallah Yoo, Jin Hee Im, Sun Ha Paek, Beomseok Jeon

**Affiliations:** 1 Department of Neurology and Movement Disorder Center, Seoul National University Hospital, Seoul National University College of Medicine, Seoul, Korea; 2 Department of Neurology, Seoul Metropolitan Government–Seoul National University Boramae Medical Center, Seoul National University College of Medicine, Seoul, Korea; 3 Department of Neurosurgery and Movement Disorder Center, Seoul National University Hospital, Seoul National University College of Medicine, Seoul, Korea; Philadelphia VA Medical Center, UNITED STATES

## Abstract

**Objectives:**

Deep brain stimulation (DBS) of the subthalamic nucleus (STN) has been reported to have a positive effect on sleep-wake disturbance in Parkinson's disease (PD). We aimed to investigate the long-term effects of STN DBS on sleep in patients with PD.

**Methods:**

Sixty-one patients with PD who had undergone bilateral STN DBS were followed for 3 years with assessments including the Parkinson’s disease sleep scale (PDSS), Epworth sleepiness scale (ESS), total sleep hours per day, Unified PD Rating Scale part I-III, Hoehn & Yahr stage, levodopa equivalent dose, quality of life measure, and depression scale measured preoperatively and at 6 months after postoperatively, and annually thereafter.

**Results:**

Among the 61 patients at baseline, 46 patients completed the last follow-up assessment. The total PDSS score significantly improved after STN DBS from baseline up to 3 years after STN DBS (79.0±30, 100.0±23.3, 98.8±23.0, 97.1±29.6, and 93.3±28.0 at baseline, 6, 12, 24, and 36 months, respectively, *p* = 0.006 for the change over time). Among the eight PDSS domains, the domains for overall quality of a night’s sleep, sleep onset and maintenance insomnia, and nocturnal motor symptoms showed significant improvement after STN DBS (*p* = 0.036, 0.029, and < 0.001, respectively, for the change over time). The total sleep hours per day were increased, but the total ESS score did not show significant change after STN DBS (*p* = 0.001 and 0.055, respectively, for the change over time). Changes in the total PDSS were associated with changes in the depression and motivation items in the Unified PD Rating Scale part I, depression scale, and quality of life measure, but those variables at baseline were not predictive of changes in the total PDSS after STN DBS.

**Conclusion:**

In the largest systematic long-term follow-up study, the improvement in subjective sleep quality after bilateral STN DBS was sustained in PD patients. Improved nocturnal sleep and nocturnal motor symptoms were correlated with an improved mood and quality of life. However, STN DBS did not reduce excessive daytime sleepiness despite reductions in antiparkinsonian medications.

## Introduction

Sleep-wake disturbance is one of the nonmotor symptoms in Parkinson’s disease (PD), which can present in the early stage of PD, even prior to the appearance of motor symptoms, and it can significantly impact the patients’ quality of life (QoL) [1–3]. Deep brain stimulation (DBS) of the subthalamic nucleus (STN) has been reported to have a positive effect on sleep-wake disturbance improving the subjective sleep quality on the Parkinson's Disease Sleep Scale (PDSS) [4–11] and also objective sleep architecture on polysomnography in PD patients [6,8,11–14]. However, little is known about the long-term effects of STN DBS on sleep because most prior studies had a follow-up of no longer than one year after surgery with a lack of detailed outcomes for sleep parameters [5,14–18]. In addition, results of the DBS effects on excessive daytime sleepiness are conflicting [5,14–18].

In this study, we aimed to investigate long-term effects of STN DBS on sleep using the PDSS [19] and the Epworth sleepiness scale (ESS) [20] by following up sixty-one PD patients who were treated with STN DBS. We hypothesized that the benefit of DBS on the subjective sleep quality might not be sustained beyond one year after DBS surgery. It would be more likely to gradually decline considering the progressive effects of PD and aging on sleep. Additionally, if the sleep parameters do change over time after STN DBS, to investigate clinical variables that may affect the patients’ sleep, we evaluated the association of the changes in the PDSS and ESS scores with the changes in the PD-associated motor and non-motor scales scores including the Unified Parkinson's Disease Rating Scale (UPDRS) score [21], Hoehn & Yahr (H&Y) stage [22], quality of life measure, and depression scale. We also investigated the predictive baseline clinical variables for changes in sleep parameters after STN DBS.

## Materials and methods

### Participants

Patients with PD who underwent bilateral STN DBS from July 2011 to October 2015 at the Movement Disorders Clinic at Seoul National University Hospital were included in this study. The surgical procedures were previously described [23]. Because patients with impaired cognitive function shown by a Mini-Mental State Examination (MMSE) score of less than 25 points, severe psychiatric or behavioral disturbances, and structural brain lesions on neuroimaging prior to DBS are not recommended for DBS [24], those patients were excluded in this study, and patients with unilateral STN DBS and severe post-DBS surgical complications were also excluded. Sixty-one patients met the inclusion criteria of this study. Patients were evaluated before DBS surgery at baseline and at 6, 12, 24, and 36 months after bilateral STN DBS during their regular visits for follow-up. Patients who did not follow-up at each visit were interviewed by telephone on their scheduled visit days in order to find out their reasons for missing the visit and to check their post-DBS condition. The study protocol was approved by the Seoul National University Hospital Institutional Review Board and followed the principles of the Declaration of Helsinki.

### Clinical evaluation

We assessed the sleep parameters which were measured by the PDSS and ESS during the regular visits. The PDSS questionnaire consists of 15 items with scores from 0 to 10 for each symptom (0, severe symptoms; 10, absence of symptoms; overall scores range from 0 to 150) [19], for which the items are classified into eight domains as follows: overall quality of a night’s sleep (item 1), sleep onset and maintenance insomnia (items 2 and 3), nocturnal restlessness (items 4 and 5), nocturnal psychosis (items 6 and 7), nocturia (items 8 and 9), nocturnal motor symptoms (items 10–13), sleep refreshment (item 14), and daytime dozing (item 15). The ESS questionnaire consists of 8 items with scores from 0 to 4 for each symptom (0, absence of symptoms; 4, severe symptoms; overall scores range from 0 to 24) [20]. In addition, total sleep hours per day including daytime naps were assessed by use of patients’ self-reports.

We also evaluated the clinical variables at each visit, which could potentially affect the sleep parameters in PD. The variables included the UPDRS part I-III score [21] and H&Y stage [22] in the on and off anti-parkinsonian medication condition with the DBS turned on condition, total daily levodopa equivalent dose (total LED) calculated as previously described [25], quality of life score measured by the Schwab and England Activities of Daily Living Scale (ADL) [26] in the on and off medication condition and the 36-Item Short Form Health Survey (SF-36) physical and mental health score [27], and depression score measured by the Beck depression inventory (BDI) [28].

### Statistical analysis

We used the linear mixed model to examine the changes over time from baseline to three years post-STN DBS (each visit at 6 months, 1, 2, and 3 years post-STN DBS), of which analysis considers individual differences in interval time by adding random effects, after being tested for a normal distribution using the Shapiro-Wilk test. Post-hoc t-tests with Bonferroni correction for multiplicity were used to assess the difference in changes between the visits. Changes in the total PDSS and total ESS scores from baseline to three years post-DBS were correlated with the clinical variables (the UPDRS part I-III, H&Y stage, total LED, ADL, SF-36 physical and mental health, and BDI) at baseline and their changes from baseline to three years post-DBS using partial correlation adjusted for age, gender, and disease duration. We compared the baseline characteristics of the patients who were regularly followed and completed all of visits and the dropped-out patients by Mann–Whitney U test for continuous variables and Chi-squared test for categorical variables. SPSS 21.0 (SPSS Inc., Chicago, IL, USA) was used for the statistical analysis with the significance set at 0.05 (two-tailed).

## Results

### Demographics and PD-associated characteristics of the PD patients with STN DBS

The baseline demographics of the sixty-one patients before DBS surgery are shown in Table 1. The mean patient age at STN DBS was 61.2±8.2 years, and the mean disease duration at the time of STN DBS was 17.3±5.8 years. The mean UPDRS part III score and H&Y stage in the on and off anti-parkinsonian medication condition were 22.8±11.1 and 44.4±14.1, and 2.5±0.7 and 3.2±0.8, respectively. Among the sixty-one patients at baseline, fifty-eight patients completed a 6-month evaluation, 55 completed a 12-month evaluation, 45 completed a 24-month evaluation, and 46 completed a 36-month evaluation. Thirty-eight patients completed those total five visits from baseline to the last follow-up at 36-month. In comparison of the baseline characteristics between the 38 patients and the remaining patients, there were no differences in demographics and all of PD-associated characteristics except for the UPDRS part II score in the on medication condition shown in Table 2. On the telephone follow-up for the dropped-out patients at each visit, there were no reports associated with post-DBS complications or other significant changes in their ADL after STN DBS.

**Table 1:** Demographics and PD-associated characteristics in PD patients with STN DBS.

Variables	PD patients, N = 61
Age at DBS, yr.	
Mean (SD)	61.2 (8.2)
Gender, n (%)	
Male	27 (55.7)
Female	34 (44.3)
Disease duration, yr.	
Mean (SD)	17.3 (5.8)
Age of PD onset, yr.	
Mean (SD)	43.9 (8.5)
UPDRS score & subscores, mean (SD)	
UDPRS part I-III total score		
On / Off medication	36.0 (16.2) / 74.9 (20.5)
UPDRS part I	
On / Off medication	1.9 (2.0) / 4.0 (3.4)
UPDRS part II	
On / Off medication	11.5 (8.6) / 26.5 (9.3)
UPDRS part III	
On / Off medication	22.8 (11.1) / 44.4 (14.4)
H&Y stage, mean (SD)	
On / Off medication	2.5 (0.7) / 3.2 (0.8)
Total LED, mg/day	
Mean (SD)	1662.6 (744.2)
ADL (%)	
On / Off medication	78.9 (19.6) / 43.4 (23.3)
SF-36 score & subscores, mean (SD)	
SF-36 total (physical & mental) score	304.9 (145.6)
SF-36 physical health	145.2 (67.5)
SF-36 mental health	159.6 (91.6)
BDI	
Mean (SD)	21.7 (10.1)
MMSE	
Mean (SD)	28.0 (1.6)

PD, Parkinson’s disease; UPDRS, Unified Parkinson’s disease rating scale; H&Y, Hoehn & Yahr; LED, Levodopa equivalent dose; ADL, Activities of daily living; SF-36, 36-Item Short Form Health Survey; BDI, Beck depression inventory; MMSE, Mini-mental state examination.

**Table 2:** Baseline demographics and PD-associated characteristics of the patients with or without completion of all five visits in this study.

Variables	PD patients with completion of all five visits, N = 38	PD patients without completion of all five visits, N = 23	*p* values
Age at DBS, yr.			
Mean (SD)	60.2 (7.4)	62.8 (9.2)	0.161
Gender, n (%)			0.663
Male	16 (42.1)	11 (47.8)	
Female	22 (57.9)	12 (52.2)	
Disease duration, yr.			
Mean (SD)	17.2 (6.2)	17.4 (5.2)	0.788
Age of PD onset, yr.			
Mean (SD)	43.0 (8.8)	45.4 (8.0)	0.213
UPDRS score & subscores, mean (SD)			
UPDRS part I			
On / Off medication	1.9 (1.7) / 4.4 (3.5)	1.9 (2.4) / 3.4 (3.3)	0.443 / 0.218
UPDRS part II			
On / Off medication	9.5 (6.7) / 25.4 (9.6)	14.7 (10.3) / 28.3 (8.6)	0.040* / 0.204
UPDRS part III			
On / Off medication	21.5 (9.4) / 43.2 (13.6)	25.0 (13.3) / 46.4 (15.7)	0.384 / 0.352
H&Y stage, mean (SD)			
On / Off medication	2.5 (0.6) / 3.0 (0.7)	2.5 (0.8) / 3.4 (0.9)	0.265 / 0.053
Total LED, mg/day			
Mean (SD)	1644.7 (855.1)	1692.1 (528.5)	0.356
ADL (%)			
On / Off medication	82.8 (15.3) / 47.4 (23.2)	72.6 (24.3) / 40.0 (22.4)	0.115 / 0.115
SF-36 score & subscores, mean (SD)			
SF-36 total (physical & mental) score	327.6 (156.3)	267.3 (120.0)	0.214
SF-36 physical health	156.4 (73.7)	126.8 (52.1)	0.155
SF-36 mental health	171.2 (94.7)	140.5 (84.8)	0.294
BDI			
Mean (SD)	19.7 (8.4)	25.3 (12.0)	0.080
MMSE			
Mean (SD)	28.3 (1.5)	27.6 (1.8)	0.111

PD, Parkinson’s disease; UPDRS, Unified Parkinson’s disease rating scale; H&Y, Hoehn & Yahr; LED, Levodopa equivalent dose; ADL, Activities of daily living; SF-36, 36-Item Short Form Health Survey; BDI, Beck depression inventory; MMSE, Mini-mental state examination.

*p values < 0.05 by Mann–Whitney U test or Chi-squared test.

### PDSS and ESS changes after STN DBS

**Fig 1:**
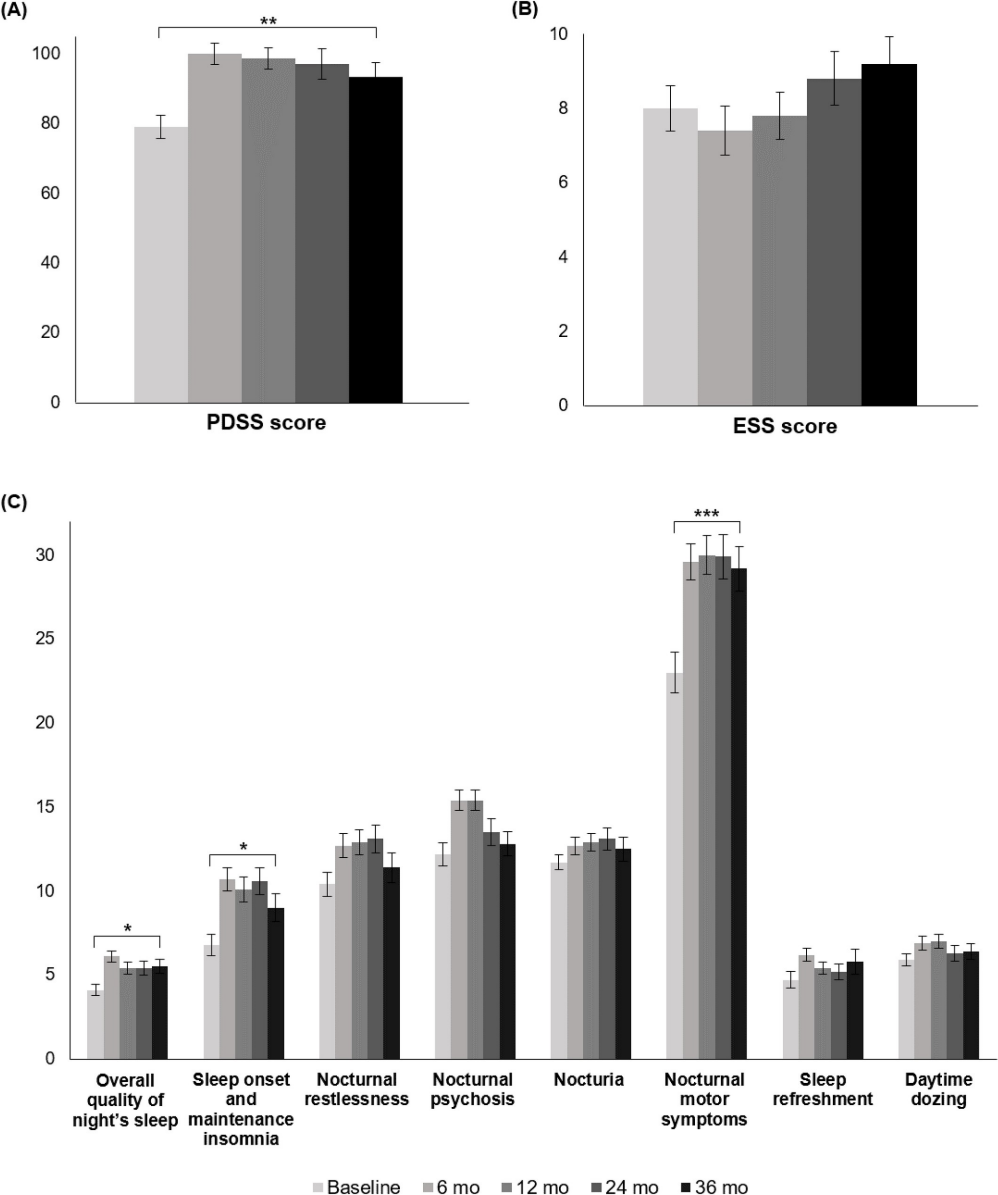
3-year follow-up changes in the PDSS and ESS scores of PD patients with STN DBS. (A) Mean PDSS total score over time. (B) Mean ESS total score over time. (C) Mean scores over time for the eight PDSS domains. *p values < 0.05, ** < 0.01, and *** < 0.001 by linear mixed model.

The total PDSS score before STN DBS was 79.0±30, and it increased after STN DBS to 100.0±23.3, 98.8±23.0, 97.1±29.6, and 93.3±28.0 at 6, 12, 24, and 36 months, respectively, which showed a significant improvement over time (*p* = 0.006 for the change over time), and compared with the baseline, the largest improvement was at 6 months as described in Table 3 and Fig 1A. Post hoc t-tests revealed significant differences between the total PDSS score at baseline and the scores at 6, 12, 24, and 36 months post-STN DBS (*p* < 0.001, < 0.001, 0.001, and 0.005, respectively). Among the eight PDSS domains, the domains scores for overall quality of a night’s sleep, sleep onset and maintenance insomnia, and nocturnal motor symptoms showed significant increases after STN DBS (*p* = 0.036, 0.029, and < 0.001, respectively, for the change over time), and compared with the baseline, the largest improvement was at 6 months shown in Table 3 and Fig 1C. In the post hoc t-test evaluation, the overall quality of a night’s sleep score at baseline and the score at 6 months post-STN DBS significantly differed (*p* < 0.001). The sleep onset and maintenance insomnia score showed differences between the baseline and 6, 12, and 24 months post-STN DBS (*p* < 0.001, 0.001, and < 0.001, respectively), and the nocturnal motor symptoms score showed differences between the baseline and 6, 12, 24, and 36 months post-STN DBS in the post hoc t-tests (all *p* < 0.001). The scores of the nocturnal restlessness, nocturnal psychosis, nocturia, sleep refreshment, and daytime dozing tended to increase after STN DBS but not to a statistically significant degree (*p* = 0.284, 0.894, 0.335, 0.321, and 0.809, respectively, for the change over time). The total sleep hours per day on the patients’ reports showed an increase after STN DBS (*p* = 0.001 for the change over time) shown in Table 3. In the post hoc t-test, the differences were found between the baseline and 6, 24, and 36 months post-STN DBS (*p* = 0.002, 0.005, and 0.003, respectively).

**Table 3:** Sleep parameters as PDSS and ESS scores and sleep hour per day over time in PD patients with STN DBS.

Variable	Baseline	6 mo.	12 mo.	24 mo.	36 mo.	*p* values
	(n = 61/61)	(n = 58/61)	(n = 55/61)	(n = 45/61)	(n = 46/61)	Change over time
PDSS total score, mean (SD)	79.0 (30.0)	100.0 (23.3)	98.8 (23.0)	97.1 (27.6)	93.3 (28.0)	0.006*
8 PDSS subscore, mean (SD)						
1. Overall quality of night’s sleep	4.1 (2.6)	6.1 (2.5)	5.4 (2.6)	5.4 (2.8)	5.5 (2.8)	0.036*
2. Sleep onset and maintenance insomnia	6.8 (4.9)	10.7 (5.4)	10.1 (5.5)	10.6 (5.3)	9.0 (5.7)	0.029*
3. Nocturnal restlessness	10.4 (5.6)	12.7 (5.6)	12.9 (5.6)	13.1 (5.6)	11.4 (6.0)	0.284
4. Nocturnal psychosis	12.2 (5.4)	15.4 (4.5)	15.4 (4.5)	13.5 (5.2)	12.8 (4.9)	0.894
5. Nocturia	11.7 (3.6)	12.7 (4.0)	12.9 (4.0)	13.1 (4.5)	12.5 (4.9)	0.335
6. Nocturnal motor symptoms	23.0 (9.5)	29.6 (8.1)	30.0 (8.5)	29.9 (9.0)	29.2 (8.9)	<0.001*
7. Sleep refreshment	4.7 (3.8)	6.2 (3.0)	5.4 (2.7)	5.2 (3.1)	5.8 (5.0)	0.321
8. Daytime dozing	5.9 (2.9)	6.9 (3.2)	7.0 (3.1)	6.3 (3.1)	6.4 (3.3)	0.809
ESS total score, mean (SD)	8.0 (4.8)	7.4 (5.1)	7.8 (4.6)	8.8 (4.8)	9.2 (5.0)	0.055
Total sleep/day, h	6.4 (1.9)	7.5 (1.5)	7.2 (1.5)	7.7 (2.7)	7.7 (2.1)	0.001*

PDSS, Parkinson’s disease sleep scale; ESS, Epworth sleepiness scale.

*p values < 0.05 by linear mixed model.

The total ESS score before STN DBS was 8.0±4.8, and after the surgery, it were 7.5±1.5, 7.2±1.5, 7.7±2.7, and 7.7±2.1 at 6, 12, 24, and 36 months, respectively, which were not significant changes over time (*p* = 0.055 for the change over time) as presented in Table 3 and Fig 1B. On the other hand, antiparkinsonian medications showed significant reductions over time (total LED before STN DBS = 1662.6±744.2; total LED after STN DBS = 509.5±381.1, 512.1±361.4, 578.2±389.5 and 670.7±568.7 at 6, 12, 24, and 36 months, respectively; *p* < 0.001 for the change over time).

### Association of the changes in PDSS and ESS with clinical variables after STN DBS

When we analyzed the clinical variables that could potentially affect the changes in sleep parameters from baseline to three years post-DBS, we found changes in the total PDSS score were significantly associated with the changes in the UPDRS part I score, especially for the scores of the depression and motivation items in the UPDRS part I and the BDI (*r* = -0.411, -0.465, and -0.557, *p* = 0.007, 0.002, and < 0.001, respectively) shown in Table 4. Changes in the total PDSS score were also related to the QoL showing significant associations with changes in the UPDRS part II score and SF-36 physical and mental health scores (*r* = -0.387, 0.363, and 0.485, *p* = 0.022, 0.032, and 0.003, respectively). Changes in the UPDRS part III score, H&Y stage, and total LED, however, were not associated with the changes in the total PDSS score (*p* = 0.933, 0.231 and 0.270, respectively). Meanwhile, changes in the total ESS score showed no significant associations with the changes in any of the scores for the clinical variables described in Table 4.

**Table 4:** Correlation coefficients of the changes in the PDSS and ESS scores with changes in the clinical variables.

	Δ PDSS total score	Δ ESS total score
Δ UPDRS part I score		
On medication	-0.556***	0.052
Off medication	-0.564***	-0.227
Δ UPDRS part II score		
On medication	-0.116	0.156
Off medication	-0.387*	-0.112
Δ UPDRS part III score		
On medication	0.045	-0.003
Off medication	-0.015	-0.128
Δ H&Y stage		
On medication	0.012	-0.017
Off medication	-0.208	0.129
Δ Total LED	0.192	0.101
Δ ADL score		
On medication	0.252	-0.055
Off medication	0.327	0.109
Δ SF-36 score		
Physical health score	0.363*	-0.056
Mental health score	0.485**	-0.040
Δ BDI score	-0.557***	0.147
Δ ESS total score	-0.098	-

Δ, changes from baseline to post-DBS year 3; PDSS, Parkinson’s disease sleep scale; ESS, Epworth sleepiness scale; UPDRS, Unified Parkinson’s disease rating scale; H&Y, Hoehn & Yahr; LED, Levodopa equivalent dose; ADL, Activities of daily living; SF-36, 36-Item Short Form Health Survey; BDI, Beck depression inventory.

*p values < 0.05

** < 0.01, and

*** < 0.001 by partial correlation adjusted for age, gender, and disease duration.

In the analysis for the predictive baseline clinical factor for sleep outcome after STN DBS by the partial correlation adjusting for age, gender, and disease duration, we observed no significant relationship between the changes in the total PDSS score and the baseline scores for the clinical variables.

## Discussion

In this study, we investigated the long-term effects of STN DBS on sleep-wake disturbance by three-year following up the PD patients who underwent bilateral STN DBS. Our major findings were as follows. First, STN DBS led to a sustained improvement in sleep-wake disturbance of PD patients. Specifically, STN DBS contributed to improvement in nocturnal parkinsonian motor symptoms, overall quality of a night’s sleep, and sleep onset and maintenance insomnia, and these benefits were maintained even three years after the surgery. Second, the improved sleep-wake disturbance was associated with an better mood and quality of life after STN DBS.

Our findings were consistent with previous studies that showed positive impact of DBS surgery on sleep-wake disturbance; however, most of them had a follow-up of no longer than one year, and the improvement had a decreasing tendency over time [4–10]. Based on this and the progression of neurodegenerative PD with aging, we hypothesized that the initial improvement in sleep quality was followed by deterioration over one year after STN DBS. Contrary to the hypothesis, our data showed that the total PDSS score at baseline and those scores at all post-STN DBS visits (6 months, 1, 2, and 3 years post-STN DBS) differed, which meant improved sleep quality was prolonged over time. Although the largest improvement in the total PDSS score was at post-DBS 6 months and it seemed to gradually decline afterwards, there was no difference among those scores at 1, 2, and 3 years post-STN DBS. However, excessive daytime sleepiness assessed by the ESS did not show a significant change in our study. Previous studies of DBS effects on excessive daytime sleepiness in PD patients have shown inconsistent results; some studies reported a significant improvement in daytime sleepiness after STN DBS with reduced antiparkinsonian medication [5,14,18] whereas other studies failed to show any changes in daytime sleepiness [15–17]. In the present study, despite the significant reduction in antiparkinsonian medications, the total ESS score and the subscore for daytime dozing in the PDSS did not differ after STN DBS. In addition, changes in the total ESS score were not associated with the increased changes in the total PDSS score and the decreased changes in the total LED from baseline to three years post-DBS. However, total sleep hours per day on the patients’ reports were increased after STN DBS in our study. This is probably due to increased night-time sleep given that nocturnal sleep quality was significantly improved after STN DBS.

It remains unknown which factors contribute to sleep after DBS surgery. In our study, sleep parameter for nocturnal motor symptoms indicated by the PDSS was significantly improved after STN DBS, and the increased PDSS score was correlated with a decreased depressive mood and an increased motivation reflected by the UPDRS part I and BDI. Better motor symptoms after STN DBS may contribute to the improvement in sleep quality, and the improved sleep quality after DBS surgery may lead to improvement in mood disorders in PD patients. Another possibility is that STN DBS has a direct effect on sleep physiology independent of the improvement in the motor and non-motor symptoms. Because the STN has important reciprocal connections with the sleep-wake modulating structures, it may affect sleep [18,29,30]. Otherwise, as multifactorial effects, several factors including the direct effect of STN DBS and motor and non-motor symptoms may affect sleep quality after STN DBS.

Our study has some limitations. This is a retrospective observational study performed in a single center. Among sixty-one patients at baseline, 75.4% of the patients were followed from baseline to three years after STN DBS, which raises the possibility of a dropout bias. However, on the telephone follow-up for the dropped-out patients, we could not find substantial changes in their post-DBS daily living. Furthermore, the baseline characteristics between the dropped-out patients and those who completed all visits in our study did not significantly differ. Another limitation is that we did not include a control group and measured the subjective assessment of sleep parameters which lack validation with the objective measures. Lastly, sleep disorders such as obstructive sleep apnea, restless leg syndrome, rapid eye movement sleep behavior disorder, and other than anti-parkinsonian medication-induced insomnia, which could possibly affect sleep, were not evaluated.

In conclusion, this is the largest systematic long-term follow-up study of bilateral STN DBS contribution to sleep in PD patients. Our results demonstrated that bilateral STN DBS contributed to sustained improvement in overall nocturnal sleep quality, and getting the better night's sleep was correlated with better mood and quality of life after the DBS surgery. Our novel findings provide convincing evidence for the beneficial effect of bilateral STN DBS on sleep and related factors in PD patients.

## Supporting information

S1 Dataset Sleep parameters data, correlation and post hoc t-test analysis. (XLSX)
